# Supplementary material for: Genetic analyses identify pleiotropy and causality for blood proteins and highlight Wnt/β-catenin signalling in migraine
Source: Nat Commun. 2022 May 11;13:2593. doi: 10.1038/s41467-022-30184-z (PMC9095680; doi:10.1038/s41467-022-30184-z)
Supplement: Supplementary file 1 — Supplementary Information [file 41467_2022_30184_MOESM1_ESM.pdf]

Supplementary information for:

Genetic analyses identify pleiotropy and causality for blood proteins and highlight Wnt/β-catenin signalling in migraine

Hamzeh M Tanha[1*], The International Headache Genetics Consortium[2], Dale R Nyholt[1*]

[1] School of Biomedical Sciences, Faculty of Health, and Centre for Genomics and Personalised Health, Queensland University of Technology (QUT), Brisbane, Australia

[2] Full list of members appears at the end.

*Correspondence to: Hamzeh M Tanha hamzeh.mesriantanha@hdr.qut.edu.au and Dale R Nyholt d.nyholt@qut.edu.au; School of Biomedical Sciences, Faculty of Health, and Centre for Genomics and Personalised Health, Queensland University of Technology (QUT), 60 Musk Avenue, Kelvin Grove QLD 4059, Brisbane, Australia

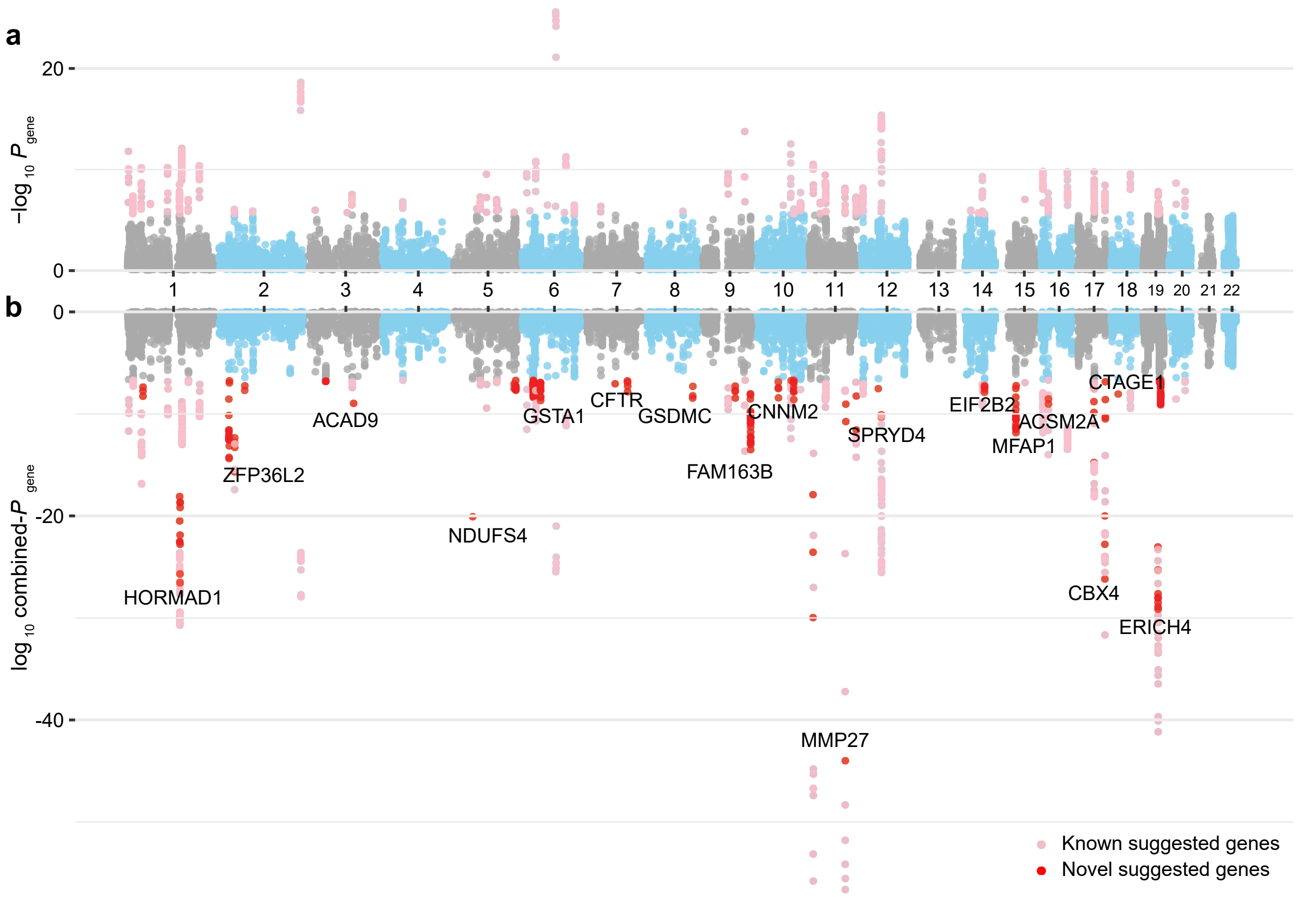

Supplementary Fig 1. Manhattan plot of genes involved in migraine identified from migraine GWAS and combined analyses. **a** MAGMA results from migraine GWAS identified 494 genes (pink) associated with migraine risk at $P_{gene} \leq 0.05/18,236$. **b** Combining MAGMA P_{gene} values from migraine GWAS and the 15 identified proteins (Figure 2c) identified 651 genes (pink and red) associated with both migraine risk and blood levels of the protein(s) at combined-$P_{gene} \leq 0.05/291,776$, of which 254 genes are novel (red). Note, unadjusted (combined-)P_{gene} are shown.

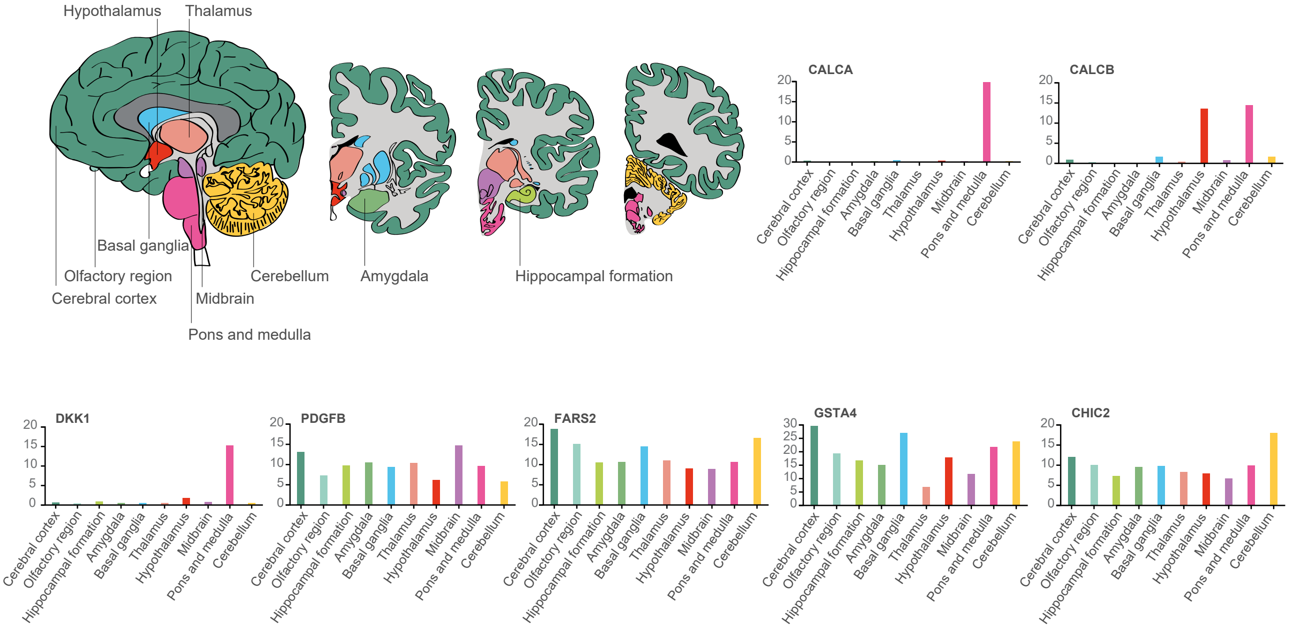

Supplementary Figure 2. The RNA expression levels of the migraine therapeutic target genes (*CALCA* and *CALCB*) and five identified proteins with a causal effect on migraine (*DKK1*, *PDGFB*, *GSTA4*, *FARS2* and *CHIC2*) across ten brain regions. The brain schematics and bar plots were obtained from the brain atlas of the Human Protein Atlas (HPA, version 20.1, https://www.proteinatlas.org/) (1). Notably, RNA expression levels are consensus normalized expression (NX) levels generated by combining data from GTEx and FANTOM5. The colours represent different brain regions, and the bars show NX levels.

Supplementary Table 1. Reported details on the six separate studies that have provided GWAS summary statistics for 4,625 blood proteins.

First author Year (Sample size / number of reported proteins in the paper)	Details provided in the paper
**Kettunen 2016 (24,925 / 4) – Serum/plasma proteins**	Population: 14 cohorts from Europe<br>Biofluid: Mostly fasting serum/plasma platform<br>Trait kind: 4 proteins in addition to 119 metabolites [not included in this study but analysed elsewhere (2)]<br>Platform: NMR platform<br>Identified loci: 62 loci were significantly associated with at least one measure.<br>Genome assembly: Human genome build 39 (19?)<br>Association test: "All metabolites/proteins were first adjusted for age, sex, time from last meal, if applicable, and ten first principal components from genomic data and the resulting residuals were transformed to normal distribution by inverse rank-based normal transformation."<br>Imputation: 1000 Genomes Project March 2012 version<br>Publication: (3)<br>GWAS S-S source: http://www.computationalmedicine.fi/data.php?dataset=NMR_GWAS<br>Number of GWAS files: 4
**Sun 2018 (3,301 / 2,994) – Plasma proteome**	Population: Interval study (UK healthy blood donors)<br>Biofluid: Plasma<br>Trait kind: Proteome<br>Platform: Multiplexed, aptamer-based approach (SOMAscan assay) were used to measure the relative concentrations of 3,622 plasma proteins or protein complexes assayed using 4,034 modified aptamers ('SOMAmer reagents', hereafter referred to as SOMAmers).<br>Identified loci: 1,927 genetic associations with 1,478 proteins.<br>Genome assembly: GRCh37<br>Association test: "Log-transformed protein levels were adjusted in a linear regression for age, sex, duration between blood draw and processing and the first three principal components of ancestry from multi-dimensional scaling. The protein residuals from this linear regression were then rank-inverse normalized and used as phenotypes for association testing. Simple linear regression using an additive genetic model was used to test genetic associations."<br>Imputation: A combined 1000 Genomes Phase 3-UK10K reference panel<br>Publication: (4)<br>GWAS S-S source: http://www.phpc.cam.ac.uk/ceu/proteins/<br>Number of files: 3,283 SOMAmers files that used to assay 2,994 proteins
**Ahola-Olli 2017 (8,293 / 41) – Blood cytokine proteins**	Population: The Cardiovascular Risk in Young Finns Study (YFS), FINRISK1997, and FINRISK2002.<br>Biofluid: Plasma/Serum<br>Trait kind: 41 cytokines<br>Platform: Immuno-assay for cytokines and growth factors<br>Identified loci: 27 genome-widely significant loci for one or more cytokines.<br>Genome assembly: Human genome build 37.<br>Association test: "The transformed phenotypes were adjusted for age, sex, body mass index, and the first ten genetic principal components by calculating residuals of linear regression model."<br>Imputation: 1000 Genomes Project September 2013 release<br>Publication: (5)<br>GWAS S-S source: http://www.computationalmedicine.fi/data#Cytokine_GWAS<br>Number of files: 41
**Folkersen 2017 (3,394 / 83) – Plasma CVD proteins**	Population: The IMPROVE European subjects.<br>Biofluid: Plasma<br>Trait kind: CVD proteins<br>Platform: Immuno-assay for proteins implicated in cardiovascular diseases<br>Identified loci: 79 loci.<br>Genome assembly: ?<br>Association test: "Standardized residuals for each of the 83 plasma proteins were calculated using a linear model adjusting for age, sex, recruitment centre, protein analysis batch, smoking, diabetes and hypertension at baseline."<br>Imputation: 1000 genomes CEU v3

**First author Year (Sample size / number of reported proteins in the paper)**	**Details provided in the paper**
	Publication: (6)<br>GWAS S-S source: ftp://ftp.ebi.ac.uk/pub/databases/gwas/summary_statistics/FolkersenL_28369058_GCST009731<br>Number of files: 83
**Suhre 2017 (1,335 / 1,124) – Plasma proteome**	Population: KORA (the general population living in the region of Augsburg, southern Germany)<br>Biofluid: Plasma<br>Trait kind: Proteome<br>Platform: SOMAscan platform<br>Identified loci: 539 associations between protein levels and gene variants.<br>Genome assembly: GRCh37.p13, Ensembl version 82, 1000-Genomes (phase 3, version 5) data.<br>Association test: "Linear models, inverse-normalized probe levels, and covariates (age, gender and body mass index) were used for association test."<br>Imputation: 1000 Genomes project database.<br>Publication: (7)<br>GWAS S-S source: http://metabolomics.helmholtz-muenchen.de/pgwas/index.php?task=download<br>Number of files: 1,124
**Folkersen 2020 (30,931 / 90) – Plasma CVD proteins**	Population: SCALLOP consortium, including 13 studies.<br>Biofluid: Plasma<br>Trait kind: CVD proteins<br>Platform: Immuno-assay for proteins implicated in cardiovascular diseases<br>Identified loci: 451 pQTLs for 85 proteins.<br>Genome assembly: ?<br>Association test: "METAL meta-analysis."<br>Imputation: Data imputed to the 1000 Genomes Project phase 3 reference or later or to the Haplotype Reference Consortium (HRC) reference.<br>Publication: (8)<br>GWAS S-S source: http://www.scallop-consortium.com/<br>Number of files: 90

Supplementary References

1. Uhlen M, Karlsson MJ, Zhong W, Tebani A, Pou C, Mikes J, et al. A genome-wide transcriptomic analysis of protein-coding genes in human blood cells. Science. 2019;366(6472).
2. Tanha HM, Sathyanarayanan A, Nyholt DR. Genetic overlap and causality between blood metabolites and migraine. Am J Hum Genet. 2021;108(11):2086-98.
3. Kettunen J, Demirkan A, Würtz P, Draisma HH, Haller T, Rawal R, et al. Genome-wide study for circulating metabolites identifies 62 loci and reveals novel systemic effects of LPA. Nat Commun. 2016;7:11122.
4. Sun BB, Maranville JC, Peters JE, Stacey D, Staley JR, Blackshaw J, et al. Genomic atlas of the human plasma proteome. Nature. 2018;558(7708):73-9.
5. Ahola-Olli AV, Würtz P, Havulinna AS, Aalto K, Pitkänen N, Lehtimäki T, et al. Genome-wide Association Study Identifies 27 Loci Influencing Concentrations of Circulating Cytokines and Growth Factors. Am J Hum Genet. 2017;100(1):40-50.
6. Folkersen L, Fauman E, Sabater-Lleal M, Strawbridge RJ, Frånberg M, Sennblad B, et al. Mapping of 79 loci for 83 plasma protein biomarkers in cardiovascular disease. PLoS Genet. 2017;13(4):e1006706.
7. Suhre K, Arnold M, Bhagwat AM, Cotton RJ, Engelke R, Raffler J, et al. Connecting genetic risk to disease end points through the human blood plasma proteome. Nat Commun. 2017;8:14357.
8. Folkersen L, Gustafsson S, Wang Q, Hansen DH, Hedman Å K, Schork A, et al. Genomic and drug target evaluation of 90 cardiovascular proteins in 30,931 individuals. Nat Metab. 2020;2(10):1135-48.

International Headache Genetics Consortium members ordered alphabetically

Verneri Anttila[1,2,3], Ville Artto[4], Andrea C Belin[5], Anna Bjornsdottir[6], Gyda Bjornsdottir[7], Dorret I Boomsma[8], Sigrid Børte[9,10,11], Mona A Chalmer[12], Daniel I Chasman[13,14], Bru Cormand[15], Ester Cuenca-Leon[16], George Davey-Smith[17], Irene de Boer[18], Martin Dichgans[19,20], Tonu Esko[21], Tobias Freilinger[22,23], Padhraig Gormley[24], Lyn R Griffiths[25], Eija Hämäläinen[26], Thomas F Hansen[12,27], Aster VE Harder[18,28], Heidi Hautakangas[26], Marjo Hiekkala[29], Maria G Hrafnsdottir[30], M. Arfan Ikram[31], Marjo-Riitta Järvelin[32,33,34,35], Risto Kajanne[26], Mikko Kallela[4], Jaakko Kaprio[26], Mari Kaunisto[29], Lisette JA Kogelman[12], Espen S Kristoffersen[36,37,38], Christian Kubisch[39], Mitja Kurki[40], Tobias Kurth[41], Lenore Launer[42], Terho Lehtimäki[43], Davor Lessel[39], Lannie Ligthart[8], Sigurdur H Magnusson[7], Rainer Malik[19], Bertram Müller-Myhsok[44], Carrie Northover[45], Dale R Nyholt[46], Jes Olesen[12], Aarno Palotie[26,47], Priit Palta[26], Linda M Pedersen[48], Nancy Pedersen[49], Matti Pirinen[26,50,51], Danielle Posthuma[52], Patricia Pozo-Rosich[53], Alice Pressman[54], Olli Raitakari[55,56,57], Caroline Ran[5], Gudrun R Sigurdardottir[6], Hreinn Stefansson[7], Kari Stefansson[7], Olafur A Sveinsson[30], Gisela M Terwindt[18], Thorgeir E Thorgeirsson[7], Arn MJM van den Maagdenberg[18,28], Cornelia van Duijn[58], Maija Wessman[29,26], Bendik S Winsvold[48,9,59], John-Anker Zwart[48,9,10]

[1]Analytical and Translational Genetics Unit, Department of Medicine, Massachusetts General Hospital and Harvard Medical School, Boston, Massachusetts, USA; [2]Program in Medical and Population Genetics, Broad Institute of MIT and Harvard, Cambridge, Massachusetts, USA; [3]Stanley Center for Psychiatric Research, Broad Institute of MIT and Harvard, Cambridge, Massachusetts, USA; [4]Department of Neurology, Helsinki University Central Hospital, Helsinki, Finland; [5]Department of Neuroscience, Karolinska Institutet, Stockholm, Sweden; [6]Neurology private practice, Laeknasetrid, Reykjavik, Iceland; [7]deCODE genetics/Amgen Inc., Reykjavik, Iceland; [8]Netherlands Twin Register, Department of Biological Psychology, Vrije Universiteit, Amsterdam, the Netherlands; [9]K.G. Jebsen Center for Genetic Epidemiology, Department of Public Health and Nursing, Faculty of Medicine and Health Sciences, Norwegian University of Science and Technology, Trondheim, Norway; [10]Institute of Clinical Medicine, Faculty of Medicine, University of Oslo, Oslo, Norway; [11]Research and Communication Unit for Musculoskeletal Health, Department of Research, Innovation and Education, Division of Clinical Neuroscience, Oslo University Hospital, Oslo, Norway; [12]Danish Headache Center, Department of Neurology, Copenhagen University Hospital, Copenhagen, Denmark; [13]Department of Medicine, Division of Preventive Medicine, Brigham and Women's Hospital, Boston, Massachusetts, USA; [14]Harvard Medical School, Boston, Massachusetts, USA; [15]Department of Genetics, Spain Centre for Biomedical Network Research on Rare Diseases, University of Barcelona, Barcelona, Spain; [16]Pediatric Neurology Research Group, Vall d'Hebron Research Institute, Barcelona, Spain; [17]University of Bristol/Medical Research Council Integrative Epidemiology Unit, University of Bristol, Bristol, UK; [18]Department of Neurology, Leiden University Medical Centre, Leiden, the Netherlands; [19]Institute for Stroke and Dementia Research, University Hospital, LMU Munich, Munich, Germany; [20]Munich Cluster for Systems Neurology, Munich, Germany; [21]Estonian Biobank Registry, the Estonian Genome Center, University of Tartu, Tartu, Estonia; [22]Department of Neurology, Klinikum Passau, Passau, Germany; [23]Department of Neurology and Epileptology, Hertie Institute for Clinical Brain Research, University of Tuebingen, Tuebingen, Germany; [24]GSK Inc., Cambridge, Massachusetts, USA; [25]Centre for Genomics and Personalised Health, Queensland University of Technology, Brisbane, Queensland, Australia; [26]Institute for Molecular Medicine Finland, Helsinki Institute of Life Science, University of Helsinki, Helsinki, Finland; [27]Novo Nordic Foundation Center for Protein Research, Copenhagen University, Copenhagen, Denmark; [28]Department of Human Genetics, Leiden University Medical Centre, Leiden, the Netherlands; [29]Folkhälsan Research Center, Helsinki, Finland; [30]Landspitali University Hospital, Reykjavik, Iceland; [31]Department of Epidemiology, Erasmus University Medical Center, Rotterdam, the Netherlands; [32]Department of Epidemiology and Biostatistics, MRC-PHE Centre for Environment and Health, School of Public Health, Imperial College

London, London, UK; [33]Center for Life Course Health Research, Faculty of Medicine, University of Oulu, Oulu, Finland; [34]Unit of Primary Health Care, Oulu University Hospital, OYS, Oulu, Finland; [35]Department of Life Sciences, College of Health and Life Sciences, Brunel University London, London, UK; [36]Research and Communication Unit for Musculoskeletal Health, Department of Research, Innovation and Education, Division of Clinical Neuroscience, Akershus University Hospital and University of Oslo, Oslo, Norway; [37]Department of General Practice, Institute of Health and Society, University of Oslo, Oslo, Norway; [38]Department of Neurology, Akershus University Hospital, Lørenskog, Norway; [39]Institute of Human Genetics, University Medical Center Hamburg-Eppendorf, Hamburg, Germany; [40]Psychiatric and Neurodevelopmental Genetics Unit, Department of Medicine, Massachusetts General Hospital, Boston, Massachusetts, USA; [41]Institute of Public Health, Charité – Universitätsmedizin, Berlin; [42]Laboratory of Epidemiology and Population Sciences, Intramural Research Program, National Institute on Aging, Bethesda, Maryland, USA; [43]Department of Clinical Chemistry, Fimlab Laboratories, and Finnish Cardiovascular Research Center - Tampere, Faculty of Medicine and Health Technology, Tampere University, Tampere, Finland; [44]Max Planck Institute of Psychiatry, Munich, Germany; [45]23&Me Inc., Mountain View, California, USA; [46]School of Biomedical Sciences, Faculty of Health, Centre for Genomics and Personalised Health, Centre for Data Science, Queensland University of Technology, Brisbane, Queensland, Australia; [47]University of Helsinki, Helsinki, Finland; [48]Department of Research, Innovation and Education, Division of Clinical Neuroscience, Oslo University Hospital, Oslo, Norway; [49]Department of Medical Epidemiology and Biostatistics, Karolinska Institutet, Stockholm, Sweden; [50]Department of Mathematics and Statistics, University of Helsinki, Helsinki, Finland; [51]Department of Public Health, University of Helsinki, Helsinki, Finland; [52]Department of Complex Trait Genetics, Center for Neurogenomics and Cognitive Research, Neuroscience Campus Amsterdam, VU University, Amsterdam, The Netherlands; [53]Headache Unit, Neurology Department, Vall d'Hebron University Hospital, Barcelona, Spain; [54]Sutter Health, Sacramento, California, USA; [55]Centre for Population Health Research, University of Turku, Turku University Hospital, Turku, Finland; [56]Research Centre of Applied and Preventive Cardiovascular Medicine, University of Turku, Turku, Finland; [57]Department of Clinical Physiology and Nuclear Medicine, Turku University Hospital, Turku, Finland; [58]Department of Epidemiology, Erasmus University Medical Centre, Rotterdam, the Netherlands; [59]Department of Neurology, Oslo University Hospital, Oslo, Norway.
