# Supplementary material for: Genetic analyses identify pleiotropy and causality for blood proteins and highlight Wnt/β-catenin signalling in migraine
Source: Nat Commun. 2022 May 11;13:2593. doi: 10.1038/s41467-022-30184-z (PMC9095680; doi:10.1038/s41467-022-30184-z)
Supplement: Supplementary file 3 — Description of Additional Supplementary Files [file 41467_2022_30184_MOESM3_ESM.pdf]

Description of Additional Supplementary Files

File Name: Supplementary Data 1

Description: Estimated LDSC polygenic SNP heritability for 4,625 blood protein GWASs.

File Name: Supplementary Data 2

Description: Pearson correlation results between 113,251 SNP *Z* scores from the migraine GWAS and *Z* scores for the same set of SNPs from 270 blood protein GWASs.

File Name: Supplementary Data 3

Description: Gene-based MAGMA result from migraine GWAS for 494 genes associated with migraine risk at $P_{gene} \leq 0.05/18,236$.

File Name: Supplementary Data 4

Description: Gene-based combined analysis for 651 genes that are contributing to both migraine risk and alterations in blood levels of the 15 identified proteins (in pleiotropy at genes analysis) at $Combined\text{-}P_{gene} \leq 0.05/291,776$.

File Name: Supplementary Data 5

Description: GWAS-PW findings including the number of loci that fit each of the GWAS-PW four scenarios for the included 325 proteins.

File Name: Supplementary Data 6

Description: 24 pleiotropic loci across 12 chromosomes identified by GWAS-PW influencing migraine and one or more blood proteins via shared SNPs.

File Name: Supplementary Data 7

Description: The g:Profiler annotation results for the included 325 proteins.

File Name: Supplementary Data 8

Gene-based MAGMA result from migraine GWAS for the 58 genes that their proteins have pleiotropy on migraine.

File Name: Supplementary Data 9

Description: The g:Profiler annotation results for the 58 genes that their proteins have pleiotropy on migraine.

File Name: Supplementary Data 10

Description: The RNA expression levels from HPA for the 58 genes that their proteins have pleiotropy on migraine across all HPA tissues.

File Name: Supplementary Data 11

Description: The protein expression levels from HPA for the 58 proteins that have pleiotropy on migraine across all HPA tissues.
