# Supplementary material for: Genetic analyses identify pleiotropy and causality for blood proteins and highlight Wnt/β-catenin signalling in migraine
Source: Nat Commun. 2022 May 11;13:2593. doi: 10.1038/s41467-022-30184-z (PMC9095680; doi:10.1038/s41467-022-30184-z)
Supplement: Supplementary file 5 — Reporting Summary [file 41467_2022_30184_MOESM5_ESM.pdf]

nature portfolio

Corresponding author(s): Hamzeh M Tanha, Dale R Nyholt

Last updated by author(s): 25/03/2022

Reporting Summary

Nature Portfolio wishes to improve the reproducibility of the work that we publish. This form provides structure for consistency and transparency in reporting. For further information on Nature Portfolio policies, see our Editorial Policies and the Editorial Policy Checklist.

Statistics

For all statistical analyses, confirm that the following items are present in the figure legend, table legend, main text, or Methods section.

n/a	Confirmed	
☐	☒	The exact sample size (*n*) for each experimental group/condition, given as a discrete number and unit of measurement
☒	☐	A statement on whether measurements were taken from distinct samples or whether the same sample was measured repeatedly
☐	☒	The statistical test(s) used AND whether they are one- or two-sided<br>*Only common tests should be described solely by name; describe more complex techniques in the Methods section.*
☒	☐	A description of all covariates tested
☐	☒	A description of any assumptions or corrections, such as tests of normality and adjustment for multiple comparisons
☐	☒	A full description of the statistical parameters including central tendency (e.g. means) or other basic estimates (e.g. regression coefficient) AND variation (e.g. standard deviation) or associated estimates of uncertainty (e.g. confidence intervals)
☐	☒	For null hypothesis testing, the test statistic (e.g. *F*, *t*, *r*) with confidence intervals, effect sizes, degrees of freedom and *P* value noted<br>*Give P values as exact values whenever suitable.*
☒	☐	For Bayesian analysis, information on the choice of priors and Markov chain Monte Carlo settings
☒	☐	For hierarchical and complex designs, identification of the appropriate level for tests and full reporting of outcomes
☐	☒	Estimates of effect sizes (e.g. Cohen's *d*, Pearson's *r*), indicating how they were calculated

Our web collection on statistics for biologists contains articles on many of the points above.

Software and code

Policy information about availability of computer code

Data collection	No software was used.
Data analysis	The Linux command-line scripts and R functions used to format GWAS summary statistics data and to perform statistical analyses are available from the corresponding authors upon request. Software and tools can be accessed for g:Profiler/g:GOSt web-based tool (accessed Jun 2021) at https://biit.cs.ut.ee/gprofiler/gost, for GWAS-PW (v0.21) at https://github.com/joepickrell/gwas-pw, for LCV (v1.0) at https://github.com/lukejoconnor/LCV, for LDSC (v1.0.1) at https://github.com/bulik/ldsc, for LocusZoom online tool (accessed Jun 2021) at http://csg.sph.umich.edu/locuszoom for MAGMA (v1.07b) at https://ctg.cncr.nl/software/magma, for PLINK (v1.9) at https://www.cog-genomics.org/plink2, for R (v4.0.3) at https://www.r-project.org/, and for RAISS (v1.0) at https://statistical-genetics.pages.pasteur.fr/raiss/.

For manuscripts utilizing custom algorithms or software that are central to the research but not yet described in published literature, software must be made available to editors and reviewers. We strongly encourage code deposition in a community repository (e.g. GitHub). See the Nature Portfolio guidelines for submitting code & software for further information.

Data

Policy information about availability of data

All manuscripts must include a data availability statement. This statement should provide the following information, where applicable:

- Accession codes, unique identifiers, or web links for publicly available datasets
- A description of any restrictions on data availability
- For clinical datasets or third party data, please ensure that the statement adheres to our policy

To express your interest in the IHGC migraine GWAS 2016 data (except 23andMe samples), please see the details provided at http://www.headachegenetics.org/content/datasets-and-cohorts. The migraine GWAS summary statistics for the 23andMe discovery data set will be made available through 23andMe to qualified

researchers under an agreement with 23andMe that protects the privacy of the 23andMe participants. Please visit https://research.23andme.com/collaborate/#dataset-access/ for more information and to apply to access the data. All other GWAS summary statistics except migraine are publicly available (Supplementary Table 1).

Transcriptomic and proteomic data were obtained from the consensus dataset generated by the Human Protein Atlas (HPA, version 20.1, https://www.proteinatlas.org/).

Field-specific reporting

Please select the one below that is the best fit for your research. If you are not sure, read the appropriate sections before making your selection.

☒ Life sciences ☐ Behavioural & social sciences ☐ Ecological, evolutionary & environmental sciences

For a reference copy of the document with all sections, see nature.com/documents/nr-reporting-summary-flat.pdf

Life sciences study design

All studies must disclose on these points even when the disclosure is negative.

Sample size

Publicly available GWAS summary statistics for blood levels of 4,625 proteins from six studies published between March 2016 and October 2020 were obtained. The median sample size across all collected proteins GWASs was 3,301 individuals. For migraine, we used the migraine GWAS summary statistics from the 2016 report of the International Headache Genetics Consortium (IHGC) consisting of 59,674 migraine cases and 316,078 migraine-free controls.

Data exclusions

Those protein GWAS studies with a sample size < 1,000 were not resourced. Details on studies characteristics, participants, population and proteomics platforms are provided in Supplementary Table 1. We estimated polygenic SNP heritability for the 4,625 blood proteins using LDSC and then excluded GWASs with non-significant polygenic SNP heritability (one-sided $p > 0.05$), with polygenic SNP heritability > 1 (suggesting sample size noise), and duplicated GWASs. In total, 325 unique blood proteome GWASs (Table 1 in the main paper) were included for further genetic analyses. This step excluded all 41 blood cytokine proteins from Ahola-Olli et al. study (PMID: 27989323).

Where GWAS summary statistics have missing information, including rsIDs and non-effect allele, the matched human genome build (in this case GRCh37 [hg19]) was used as a reference to complete the GWAS summary statistics. To have a consistent list of SNPs for all GWASs, HapMap3 SNPs were imputed using the RAISS. Here we used 1000G LD reference to estimate Z scores of HapMap3 missing SNPs from neighbouring HapMap3 observed SNPs. To include ambiguous SNPs (G/C and A/T) in our analyses, they were removed to prior imputation and then imputed using neighbouring SNPs, making these SNPs uniformed across all studied GWASs. Standard errors for imputed SNPs were calculated using allele frequencies from 1000G and the reported sample size of the GWAS, then imputed Z scores were converted to effect sizes. To increase imputation quality, we limited our imputation to only HapMap3 common SNPs (MAF ≥ 0.01) and filtered imputed SNPs with $R2 < 0.6$. However, as imputation quality is correlated with LD scores, we carried out the LDSC and the LCV model analyses (where LD scores are used) on the original (not RAISS imputed) GWAS summary statistics.

Replication

No independent GWAS summary statistics data with a sufficiently large sample size was available for replication.

Randomization

Randomisation is not relevant to our study since we carried out genetic analyses utilising data generated in previous studies.

Blinding

The investigators were blinded during the data collection and analyses stage.

Reporting for specific materials, systems and methods

We require information from authors about some types of materials, experimental systems and methods used in many studies. Here, indicate whether each material, system or method listed is relevant to your study. If you are not sure if a list item applies to your research, read the appropriate section before selecting a response.

Materials & experimental systems

n/a	Involved in the study	
☒	☐	Antibodies
☒	☐	Eukaryotic cell lines
☒	☐	Palaeontology and archaeology
☒	☐	Animals and other organisms
☐	☒	Human research participants
☒	☐	Clinical data
☒	☐	Dual use research of concern

Methods

n/a	Involved in the study	
☒	☐	ChIP-seq
☒	☐	Flow cytometry
☒	☐	MRI-based neuroimaging

Human research participants

Policy information about studies involving human research participants

Population characteristics	GWAS summary statistics data all from European populations.
Recruitment	NA
Ethics oversight	NA

Note that full information on the approval of the study protocol must also be provided in the manuscript.
